# Dietary supplementation of benzoic acid and essential oils combination enhances intestinal resilience against LPS stimulation in weaned piglets

**DOI:** 10.1186/s40104-023-00958-6

**Published:** 2024-01-19

**Authors:** Chang Cui, Yulong Wei, Yibo Wang, Wen Ma, Xiaoyu Zheng, Jun Wang, Ziwei Ma, Caichi Wu, Licui Chu, Shihai Zhang, Wutai Guan, Fang Chen

**Affiliations:** 1 Guangdong Provincial Key Laboratory of Animal Nutrition Control, College of Animal Science, South China Agricultural University, Guangzhou, 510642 China; 2 College of Animal Science and National Engineering Research Center for Breeding Swine Industry, South China Agricultural University, Guangzhou, 510642 China; 3 Guangdong Laboratory for Lingnan Modern Agriculture, South China Agricultural University, Guangzhou, 510642 China

**Keywords:** Anti-stress, Benzoic acid, Essential oils, Intestine, LPS, Weaned piglets

## Abstract

**Background:**

The benefits of combining benzoic acid and essential oils (BAO) to mitigate intestinal impairment during the weaning process have been well established, while the detailed underlying mechanism has not been fully elucidated. Previous research has primarily focused on the reparative effects of BAO on intestinal injury, while neglecting its potential in enhancing intestinal stress resistance.

**Methods:**

In this study, we investigated the pre-protective effect of BAO against LPS-induced stress using a modified experimental procedure. Piglets were pre-supplemented with BAO for 14 d, followed by a challenge with LPS or saline to collect blood and intestinal samples.

**Results:**

Our findings demonstrated that BAO supplementation led to significant improvements in piglets' final weight, average daily gain, and feed intake/body gain ratio. Additionally, BAO supplementation positively influenced the composition of intestinal microbiota, increasing beneficial Actinobacteriota and *Alloprevotella* while reducing harmful Desulfobacterota, *Prevotella* and *Oscillospira*. Furthermore, BAO supplementation effectively mitigated oxidative disturbances and inflammatory responses induced by acute LPS challenge. This was evidenced by elevated levels of T-AOC, SOD, and GSH, as well as decreased levels of MDA, TNF-α, and IL-6 in the plasma. Moreover, piglets subjected to LPS challenge and pre-supplemented with BAO exhibited significant improvements in intestinal morphological structure and enhanced integrity, as indicated by restored expression levels of Occludin and Claudin-1 compared to the non-supplemented counterparts. Further analysis revealed that BAO supplementation enhanced the jejunal antioxidative capacity by increasing GSH-Px levels and decreasing MDA levels under the LPS challenge and stimulated the activation of the Nrf2 signaling pathway. Additionally, the reduction of TLR4/NF-κB/MAPK signaling pathways activation and proinflammatory factor were also observed in the jejunal of those piglets fed with BAO.

**Conclusions:**

In summary, our study demonstrates that pre-supplementation of BAO enhances the anti-stress capacity of weaned piglets by improving intestinal microbiota composition, reinforcing the intestinal barrier, and enhancing antioxidative and anti-inflammatory capabilities. These effects are closely associated with the activation of Nrf2 and TLR4/NF-κB/MAPK signaling pathways.

**Supplementary Information:**

The online version contains supplementary material available at 10.1186/s40104-023-00958-6.

## Introduction

Weaned piglets are typically accompanied by a multitude of weaning stressors, such as environmental and dietary changes, immature gastrointestinal tracts, and weakened immunity, all of which frequently result in intestinal dysfunction leading to diarrhea, retarded growth performance, and even high morbidity and mortality rates [1, 2]. Antibiotics have been widely used in animal diets to alleviate weaning stress by providing antibacterial effects, improving immunity, and reducing the incidence of diarrhea [3]. However, due to the serious adverse consequences of antibiotic overuse, such as animal resistance, environmental pollution, and public health concerns, an increasing number of countries have banned the use of antibiotics as feed additives for animals [4]. Therefore, the development of non-toxic and residue-free antibiotic alternatives that promote growth performance and alleviate weaning stress is critical for sustainable animal production and improving the economic benefits of the pig industry.

Benzoic acid (BA) is an organic acid (OA) with well-established antibacterial, antifungal, and preservative properties, and it has been widely used in food and feed to prevent diarrhea caused by pathogenic microorganisms, as well as to promote growth and health [5, 6]. The essential oils (EO) used in our experiment, consisting of thymol, carvacrol, eugenol, and cinnamaldehyde, are known to enhance the palatability of food, improve immune function, and promote growth performance and health due to their antioxidative and anti-inflammatory properties [7, 8]. Previous studies have demonstrated that BA has beneficial effects on intestinal development and intestinal barrier function in weaned piglets, thereby improving the growth performance [9]. In addition, dietary EO can increase the antioxidant capacity of the intestinal tract of weaned piglets, improve the intestinal morphology and reduce the diarrhea rate [10]. In recent years, amounting of studies have verified the benefits of OA and EO dietary supplementation for weaned piglets in terms of promoting their health and growth performance by improving intestinal function [11]. Subsequent investigations have demonstrated that the combination of OA and EO is more effective than individual supplementation [12]. Despite the widespread use of BA and EO mixtures (BAO) as alternative antibiotics in the diet of weaned piglets to alleviate weaning stress, the precise mechanisms underlying its protective function are not fully understood.

The intestine serves not only as an essential site for nutrient absorption and utilization, but also as a crucial immune barrier to prevent the invasion of external antigens, bacteria, and toxins into the body [13]. In healthy intestines, oxidative stress is balanced, and the intestinal barrier remains intact without triggering an inflammatory response [14]. Healthy intestines can sufficiently resist sudden acute inflammation, mitigating a series of inflammatory responses and physical damage [15]. However, unhealthy intestines are highly susceptible to inflammation and oxidative stress during periods of stress, leading to a vicious cycle of gut barrier dysfunction, inflammatory responses, and physical damage [16]. For example, intrauterine growth retardation piglets, characterized by compromised intestinal homeostasis, exhibit more severe intestinal inflammatory damage and oxidative stress during weaning stress, leading to higher incidences of diarrhea and poorer growth performance [17, 18]. Although research in recent years has established the advantageous effects of BAO on animal growth and intestinal health, it is noteworthy that most previous investigations on the impact of BAO on weaned piglets have been conducted using feeding trials as the primary experimental model. For instance, it has been reported that dietary BAO can not only improve the growth performance of weaned piglets and enhance intestinal health, but also has a alleviating effect on weaned piglets pre-infected with *Escherichia coli*, which is manifested in reducing the diarrhea rate and improving intestinal morphology [19, 20]. These previous studies primarily concentrated on the reparative effects of BAO mixtures on intestinal function because supplementation was carried out after the induction of stress-induced damage. However, no evidence has been presented to establish whether BAO mixtures could augment the intestinal anti-stress capacity, which is crucial for mitigating injury during weaning stress in piglets.

In the current study, we employed a modified experimental procedure in which weaned piglets were pre-supplemented with BAO for 14 d, followed by induction of stress using lipopolysaccharide (LPS), to assess the protective effects of BAO on plasma and intestinal antioxidant levels, immune status, and intestinal barrier integrity. This design allowed us to explore the potential of BAO in enhancing intestinal anti-stress capacity against external stimuli and elucidate the underlying mechanisms involved. Additionally, our findings have the potential to contribute to the development of nutritional strategies aimed at improving intestinal anti-stress in piglets through the use of dietary functional foods.

## Materials and methods

### Animals and feed experimental design

All animal procedures in this study were authorized by the Animal Ethics Committee of South China Agricultural University (No. 20110107-1, Guangzhou, China) on January 7, 2011 and were conducted in accordance with the Guidelines for Care and Use of Laboratory Animals of South China Agricultural University. In feed experiment, a total of 48 healthy piglets, weaned at 21 days of age (Duroc × [Landrace × Yorkshire]), were randomly divided into 2 treatments based on body weight and gender, with 4 replicates per group and 6 piglets per replicate. The piglets had been injected with swine fever vaccine, swine pseudorabies inactivated vaccine and foot-and-mouth disease vaccine. All weaned piglets are kept in a room with controlled temperature (25–30 °C) and humidity (60% ± 5%). Piglets were fed diets at 8:00, 13:00 and 18:00, three times a day. The feed experiment lasted for two weeks during which the piglets had ad libitum access to food and water. The dietary treatments were as follows: 1) corn-soybean meal basal diets (NC); 2) corn-soybean meal basal diets + 5 kg/t benzoic acid + 500 g/t essential oils (BAO). Piglets were weighed on 1 d and 14 d of the feeding experiment, and feed consumption was recorded to calculate average daily gain (ADG), average daily feed intake (ADFI), and feed intake/body gain (F/G). The calculations were performed as follows: ADG = (Total weight at the end of the experiment − total weight at the beginning of the experiment)/(number of experiment days × number of piglets); ADFI = Total feed consumption/(number of experiment days × number of piglets); F/G = ADFI/ADG.

The diets used in the study did not contain growth promoters or therapeutic antibiotics. The basal diet as showed in Additional file 1 was formulated to meet or exceed the nutrient requirements recommended by the National Research Council (NRC, 2012) [21]. The BA used in VevoVital® products had a 99% acid concentration. The EO used in the BAO treatment included thymol, eugenol, carvacrol, and cinnamaldehyde and was supplied by DSM Produtos Nutricionais Brasil SA.

### LPS challenge experiment

**Fig. 1:**
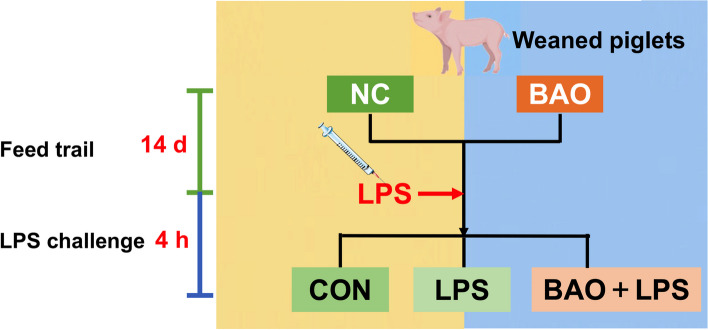
Schematic representation of the experimental design

After the two-week feed experiment, a total of 15 piglets were selected for the immunological stress experiment. The piglets were systematically allocated into three distinct groups according to their dietary regime and injection treatment (Fig. 1). The first group (CON) comprised of 5 piglets who received the basal diet and intraperitoneal injection of normal saline at a concentration of 100 μg/kg. The primary objective of this group was to serve as a control cohort for the experiment, and to provide a baseline measurement of the immune response in piglets under injection stimulating conditions. The second group (LPS) included five piglets who also received the basal diet but were intraperitoneally injected with LPS at a concentration of 100 μg/kg (*E. coli* 055:B5, Sigma). The underlying rationale of this group was to establish a model of immunological stress, and to investigate the immune response in piglets under such circumstances. Finally, the third group (BAO + LPS) comprised five piglets who received the BAO-supplemented diet and were intraperitoneally injected with LPS at a concentration of 100 μg/kg. The fundamental purpose of this group was to evaluate the potential impact of BAO supplementation on the immune response of piglets under LPS-induced stress.

### Sample collection

On the 14 days of feed experiment, 6 piglets in NC group and 6 piglets in BAO group were selected and separated them one by one into a clean crate, and then stimulated the rectum with a sterile swab. At least 5 g of feces were collected and placed directly into a sterile centrifuge tube for 16S rRNA sequencing analysis.

The 4 h post-injection of LPS or saline, 5 mL blood samples were collected from the anterior vena cava of each piglet fasted for 12 h via a blood collection needle into a heparin vacuum anticoagulant tube, following which the blood samples were allowed to stand for 0.5 h. The plasma samples were obtained via centrifugation at 3,500 × *g* for 15 min and were subsequently stored in liquid nitrogen. Subsequently, all piglets were administered with pentobarbital sodium (50 mg/kg BW) for euthanasia in the jugular vein. The weight of the heart, liver, spleen, kidney, intestine, and pancreas of each piglet was recorded to calculate the organ index. The calculation is performed as follows: organ index (g/kg) = organ weight (g)/body weight (kg). Duodenum, jejunum, and ileum were collected 2 cm and stored in 4% paraformaldehyde for morphological observation. The chyme in the jejunum tissue was washed using cold saline and then frozen in liquid nitrogen for subsequent RNA and protein measurements.

### Intestinal morphology

The intestinal specimens fixed in 4% paraformaldehyde solution were taken out, dehydrated with a gradient concentration of ethanol and embedded in paraffin blocks. The paraffin sections were 4 μm thick and stained with hematoxylin and eosin. The villus height (VH), crypt depth (CD) and VH/CD of intestinal tract were measured utilizing image processing and analysis system (NIS-Elements Viewer, Tokyo, Japan) using light microscope.

### Plasma pro-inflammatory cytokine concentration

The concentrations of TNF-α, IL-1β, IL-6, IL-12 in plasma were measured according to the manufacturer’s instructions (Mlbio, Shanghai, China). Briefly, the plasma sample was centrifuged at 3,000 × *g* for 10 min, the supernatant was collected and incubated with the corresponding antibody at 37 °C for 1 h, and then incubated with the provided chromogenic substance for 30 min before washing. Finally, the absorbance at 450 nm wavelength was measured by adding the stop solution and the concentrations in plasma were calculated by standard curve.

### Antioxidant status

A total of 70 mg of jejunal mucosa was homogenized in 4 times the volume of ice-cold normal saline solution. The supernatant was collected after centrifugation at 12,000 × *g* for 15 min and subsequently stored at −80 °C for further analysis. The concentrations of total antioxidant capacity (T-AOC), malondialdehyde (MDA), superoxide dismutase (SOD), reduced glutathione (GSH), and glutathione peroxidase (GSH-Px) in both plasma and the jejunal supernatant were determined using specific assay kits procured from Nanjing Jiancheng Bioengineering Institute.

### Western blot

The jejunal proteins were extracted by grinding and centrifugation with RIPA lysis buffer and protease inhibitor. Protein concentration in the supernatant was determined using a BCA kit (Beyotime, Shanghai, China). Then, approximately 30 μg of total protein per sample was separated by a 10% or 12% polyacrylamide gel and transferred to PVDF membrane by electrophoresis. The PVDF membranes were incubated with 6% skim milk at room temperature for 3 h, then wash with TBST for 5 times, 5 min each. After that, the PVDF membranes was blocked with corresponding primary antibody at 4 °C for 15 h. Antibodies against Claudin-1 (1:1,000, ab129119, Abcam, Cambridge, UK), ZO-1 (1:3,000, 21773-1-AP, Proteintech, Wuhan, China), Occludin (1:2,000, 27260-1-AP, Proteintech), P-JNK (1:1,000, 4688S, Cell Signaling Technology, Boston, USA), JNK (1:1,000, 9252S, Cell Signaling Technology), P-ERK(1:2,000, 9101S, Cell Signaling Technology), ERK (1:2,000, 9102S, Cell Signaling Technology), P-P38 (1:2,000, 4092S, Cell Signaling Technology), P38 (1:3,000, 66234-1-Ig, Proteintech), P-NF-κB (1:1,000, 3033S, Cell Signaling Technology), NF-κB (1:1,000, 10745-1-AP, Proteintech), P-Nrf2 (1:1,000, 381559, ZenBio, Chengdu, China), Nrf2 (1:1,000, 16396-1-AP, Proteintech), Keap1 (1:2,000, 16396-1-AP, Proteintech) and β-actin (1:2,000, bs-0061R, Bioss, Beijing, China). After washing with TBST for 5 times, the PVDF membrane was incubated with the secondary antibody goat anti rabbit (1:5,000, 511203, ZenBio) and goat anti mouse (1:50,000, 550047, ZenBio) at room temperature for 1.5 h, followed by detecting the target bands using Chemiluminescence Image Analysis System (Tanon, Shanghai, China).

### Real time quantitative PCR

About 20 mg tissue of jejunum was weighted, and total RNA was extracted according to Tissue RNA Purification kit PLUS method (EZB-RN001, EZBioscience, Roseville, USA). RNA was reversely transcribed into cDNA using Color Reverse Transcription Kit (A0010CGQ, EZBioscience) after the purity of RNA was detected by spectrophotometer. Quantitative real-time PCR was performed in a 20 µL reaction volume composed of 10 μL of 2× SYBR qPCR Mix, 0.8 μL of cDNA, 0.4 μL of Forward Primer, 0.4 μL of Reverse Primer and 8.4 μL of DEPC water using the ABI Prism 7500 Sequence Detection System (Applied Biosystems, Carlsbad, USA). The PCR reaction protocol was initial denaturation at 95 °C for 2 min, followed by 40 cycles of 95 °C for 15 s and 60 °C for 30 s. Relative expression levels of target genes were determined according to a quantitative method (2^−ΔΔCt^ method), using β-actin as a housekeeping gene to normalize all mRNA levels. The total primers used is presented in Table 1.

**Table 1:** Primer sequences used in real-time PCR

Genes	Accession	Forward primer (5'→3')	Reverse primer (5'→3')
*PBD-1*	NM_213838.1	ACCGCCTCCTCCTTGTATTCCTC	ACCGCCTCCTCCTTGTATTCCTC
*PBD-2*	NM_214442.2	ATTAACCTGCTTACGGGTCTTGGC	CCACTGTAACAGGTCCCTTCAATCC
*PBD-3*	XM_021074698.1	TCTTCTTGTTCCTGATGCCTCTTCC	GCCACTCACAGAACAGCTACCTATC
*PBD-114*	NM_001129973.1	GTACCTTGGTGGATCCTGAACGATG	AACGCCCTCTGAATGCAGCATATC
*MUC-1*	NM_001164021	CATCATCGTCCTGGTCGTC	CATCATCGTCCTGGTCGTC
*MUC-2*	EF140874	GTCCAGAAAGCCCAAGAT ACC	GTGACATCATCACTTCCTCTGAG
*MUC-4*	NM_001083931.1	GGCAACAGACGTGATCTATGAC	AGCGGCTGGCTGAAAACT
*HO-1*	NM_001004027.1	TACCGCTCCCGAATGAACAC	GTCACGGGAGTGGAGTCTTG
*NQO-1*	NM_001159613.1	GCGAGGGTCTCTGGTCCTTA	ATCACAGGTAAACTGAAGGACCC
*SOD*	NM_000454	TCCATGTCCATCAGTTTGGA	CTGCCCAAGTCATCTGGTTT
*GSH-Px*	NM_201397.2	TGGGGAGATCCTGAATTG	GATAAACTTGGGGTCGGT
*TNF-α*	NM_214022.1	CCACCAACGTTTTCCTCACT	TAGTCGGGCAGGTTGATCTC
*IL-1β*	NM_214055.1	CCAAAGAGGGACATGGAGAA	TTATATCTTGGCGGCCTTTG
*IL-6*	AF518322.1	TGGCTACTGCCTTCCCTACC	AGAGCCTGCATCAGCTCAGT
*IL-12*	NC_010458.4	CAACCCTGTGCCTTAGCAGT	AGAGCCTGCATCAGCTCAGT
*TGF-β*	NM_214015.1	GAAGCGCATCGAGGCCATTC	GGCTCCGGTTCGACACTTTC
*TLR4*	NM_001113039.1	CAACCCTGTGCCTTAGCAGT	AGAGCCTGCATCAGCTCAGT
*MyD88*	NM_001099923	CGCATGGTGGTGGTTGTT	GCCTTCTTCATCGCCTTGTATTT
*ERK*	XM_021088019.1	CAGTCTCTGCCCTCCAAGAC	GGGTAGATCATCCAGCTCCA
*JNK*	XM_001929166.6	TGGATGAAAGGGAACACACA	ATGATGACGATGGATGCTGA
*P38*	XM_021091323.1	CCCTGAGGTTCTAGCGAAGA	TCTCATCGTAGGGCTCTGCT
*NF-κB*	NM_001048232.1	CTCGCACAAGGAGACATGAA	ACTCAGCCGGAAGGCATTAT
*β-actin*	XM_021086047.1	GATCTGGCACCACACCTTCTACAAC	RTCATCTTCTCACGGTTGGCTTTGG

### 16S rRNA sequencing

MagPure Soil DNA LQ Kit (Magan, Guangzhou, China) was used to extract genomic DNA from fecal samples of piglets according to the NanoDrop 2000 guidelines (Thermo Fisher Scientific, Waltham, USA) and agarose gel electrophoresis were used to detect the concentration and purity of DNA, and the extracted DNA was stored at −20 °C for further testing. The extracted genomic DNA was utilized as a template for PCR amplification of bacterial 16S rRNA gene using primers and Takara Ex Taq (Takara, Tokyo, Japan). Universal primers (343F: 5′-TACGGRAGGCAGCAG-3′; 798R: 5′-AGGGTATCTAATCCT-3′) were used to amplify V3–V4 variable regions of 16S rRNA gene for bacterial diversity analysis. The quality of the amplicon was observed by agarose gel electrophoresis. After PCR products were purified by PCR amplification using AMPure XP beads (Agencourt, Shanghai, China), the final amplicons were measured using the Qubit dsDNA Assay kit (Thermo Fisher Scientific, Waltham, USA). The density should then be adjusted in accordance with the order. The sequence was run on an Illumina NovaSeq 6000 with 250 bp paired-end reads. (Illumina Inc., San Diego, USA; OE Biotech Company, Shanghai, China).

### Statistical analysis

Statistical analysis was performed on all data except for microbiota data using the SPSS software version 22.0 (IBM Corp., Armonk, NY, USA) to identify significant differences between the groups. The results were expressed as the means ± standard error of the mean (SEM). Correlations were conducted by Pearson correlation analysis using GraphPad Prism 9.0 (GraphPad Software, San Diego, CA, USA). A *P*-value of 0.01 < *P* < 0.05 was considered statistically significant, and *P* < 0.01 was considered to indicate an extremely significant difference between the groups.

For fecal microbiota data analysis, alpha and beta diversity analysis was performed using QIIME2 software. The microbial diversity of the samples was calculated by alpha diversity, which consisted of ACE, Chao1, goods_coverage, observed_species, PD_whole_tree, Shannon and Simpson index. The R package unweighted UniFrac distance matrix was applied to unweighted UniFrac principal coordinates analysis (PCoA) to estimate beta diversity. Then the significance of differences between groups was determined using T statistical test. The linear discriminant analysis effect size (LEfSe) was used to analyze the difference of species abundance spectrum.

## Results

### Growth performance

The results of growth performance are shown in Table 2. In comparison to the NC group, the pigs fed the BAO diet exhibited significantly greater final weight, ADG, and lower F/G ratio (*P* < 0.05). However, no significant difference was observed between the NC and BAO groups in terms of ADFI (*P* > 0.05).

**Table 2:** Effects of dietary BAO supplementation on growth performance of weaned piglets

Item	NC	BAO	SEM	*P*
Initial weight, kg	8.35	8.12	0.076	0.101
Final weight, kg	12.46^b^	13.32^a^	0.168	0.011
ADG, g	297.5^b^	372.5^a^	0.012	0.005
ADFI, g	547.5	545.0	0.005	0.730
F/G	1.863^a^	1.463^b^	0.066	0.007

Dates are presented as means ± SEM (*n* = 4)

^*ADG*Average daily gain, *ADFI*Average daily feed intake, *F/G*the ratio of feed intake to gain, *NC*Corn−soybean meal basal diets, *BAO*Corn−soybean meal basal diets+5 kg/t benzoic acid+500 g/t essential oils^

^a,b^Mean values within a row with different letters differ at *P* < 0.05

### Organ index

The organ index was obtained by dividing the organ weight by the body weight, liver organ index increased in BAO + LPS group compared to CON group (*P* < 0.05, Table 3), while the other organ measures were not statistically significant among the three groups (*P* > 0.05, Table 3).

**Table 3:** Effects of dietary BAO supplementation on organ index in piglets challenged with LPS

^Organ index^	CON	LPS	BAO + LPS	SEM	*P*
Heart	5.72	5.62	5.19	0.22	0.617
Liver	29.76^b^	32.71^ab^	35.26^a^	1.01	0.077
Spleen	2.32	2.96	2.59	0.14	0.156
Kidney	5.43	6.06	5.75	0.21	0.505
Intestine	106.46	101.31	110.04	5.15	0.810
Pancreas	1.08	1.04	0.86	0.07	0.467

Organ index (g/kg) = organ weight (g)/body weight (kg). Dates are presented as means ± SEM (*n* = 5)

*CON* Corn-soybean meal basal diets group treated with saline, *LPS* Corn-soybean meal basal diets group treated with lipopolysaccharide, *BAO* + *LPS* Corn-soybean meal basal diets + 5 kg/t benzoic acid + 500 g/t essential oils group treated with lipopolysaccharide

^a,b^Mean values within a row with different letters differ at *P* < 0.05

### Plasma antioxidant and inflammatory status

**Fig. 2:**
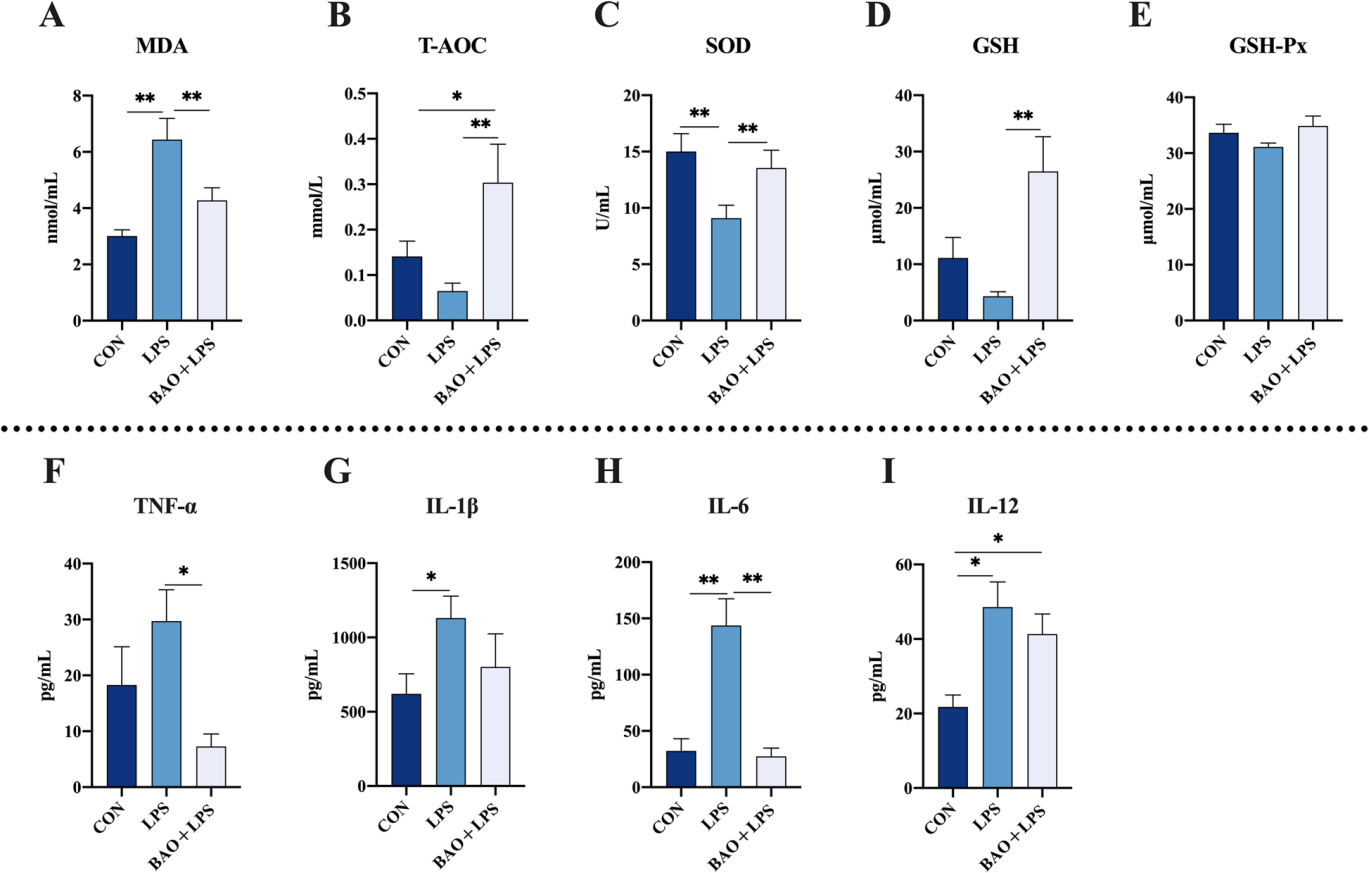
Effects of dietary BAO supplementation on plasma antioxidant capacity and inflammation levels in weaned pigs challenged with LPS. **A**–**E** The activities of MDA, T-AOC, SOD, GSH and GSH-Px. **F**–**I** The activities of TNF-α, IL-1β, IL-6 and IL-12. Dates are presented as means ± SEM (*n* = 5). ^*^P < 0.05; ^**^P < 0.01. CON, corn-soybean meal basal diets group treated with saline; LPS, corn-soybean meal basal diets group treated with lipopolysaccharide; BAO + LPS, corn-soybean meal basal diets + 5 kg/t benzoic acid + 500 g/t essential oils group treated with lipopolysaccharide

As illustrated in Fig. 2, when compared with the CON group, the level of SOD was significantly reduced in the LPS group, whereas the level of MDA was increased (*P* < 0.05). In the BAO + LPS group, there was a higher level of T-AOC, SOD, GSH, and a lower level of MDA when compared to the LPS group (*P* < 0.05). In comparison with the CON group, the T-AOC level was elevated in the BAO + LPS group (*P* < 0.05), while no significant changes were observed in MDA, SOD, GSH, and GSH-Px levels (*P* > 0.05).

In addition to the changes in plasma antioxidant status, the present study also investigated the effects of BAO on plasma inflammatory cytokines. Compared with the CON group, the LPS group had significantly increased IL-1β, IL-6, and IL-12 concentrations in plasma (*P* < 0.05). However, in the BAO + LPS group, a lower level of TNF-α and IL-6 were found compared to the LPS group (*P* < 0.05). In addition, the activity of IL-12 was upregulated in the BAO + LPS group compared with the CON group (*P* < 0.05), but TNF-α, IL-1β, and IL-6 were not significantly different between the two groups (*P* > 0.05). These findings suggest that BAO may have a protective effect against LPS-induced oxidative stress and inflammation.

### Intestinal morphology

**Fig. 3:**
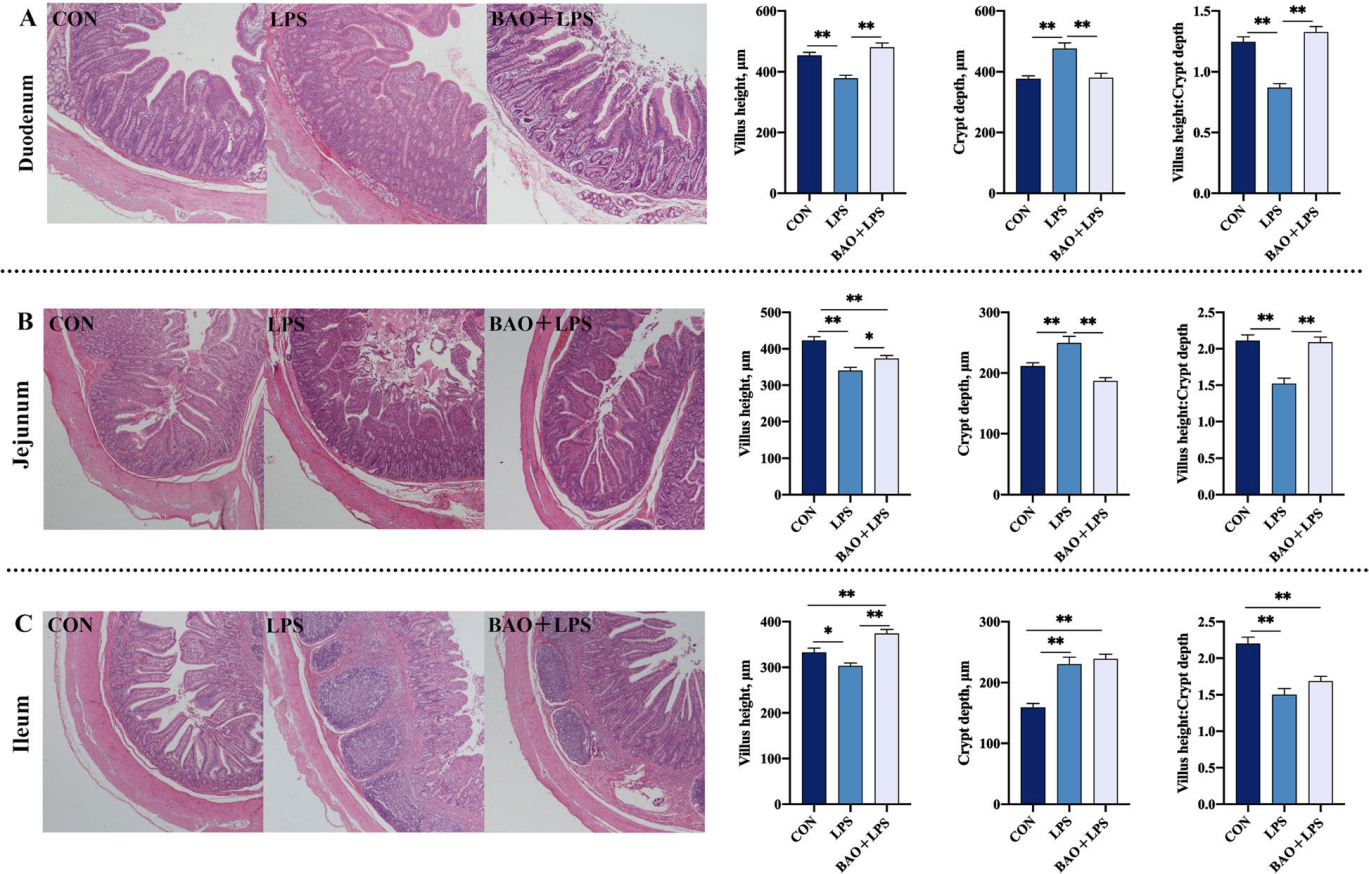
Effects of dietary BAO supplementation on intestinal morphology in weaned pigs challenged with LPS. **A** Duodenal morphology, villus height, crypt depth, ratio of villus height to crypt depth. **B** Jejunal morphology, villus height, crypt depth, ratio of villus height to crypt depth. **C** Ileal morphology, villus height, crypt depth, ratio of villus height to crypt depth. Dates are presented as means ± SEM (*n* = 5). ^*^P < 0.05; ^**^P < 0.01. CON, corn-soybean meal basal diets group treated with saline; LPS, corn-soybean meal basal diets group treated with lipopolysaccharide; BAO + LPS, corn-soybean meal basal diets + 5 kg/t benzoic acid + 500 g/t essential oils group treated with lipopolysaccharide

Compared to the CON group, both the LPS and BAO + LPS groups exhibited varying degrees of intestinal villi morphology damage, including incomplete apex structure. Specifically, the LPS group showed a significant reduction in VH and VH/CD in the duodenum, jejunum, and ileum, while exhibiting a significant increase in CD in these regions (*P* < 0.05, Fig. 3). Meanwhile, the BAO + LPS group exhibited a decrease in VH in the jejunum and ileum, an increase in CD in the ileum, and a decrease in VH/CD in the ileum (*P* < 0.05, Fig. 3). In addition, BAO supplementation significantly mitigated the intestinal structural damage caused by LPS, leading to more complete and smoother villi in the BAO + LPS group compared to the LPS group (Fig. 3). The VH of the duodenum, jejunum, and ileum in the BAO + LPS group was higher than that in the LPS group, while the CD of the duodenum and jejunum was lower, and the VH/CD of the duodenum and jejunum was also lower (*P* < 0.05, Fig. 3).

### Intestinal barrier function

**Fig. 4:**
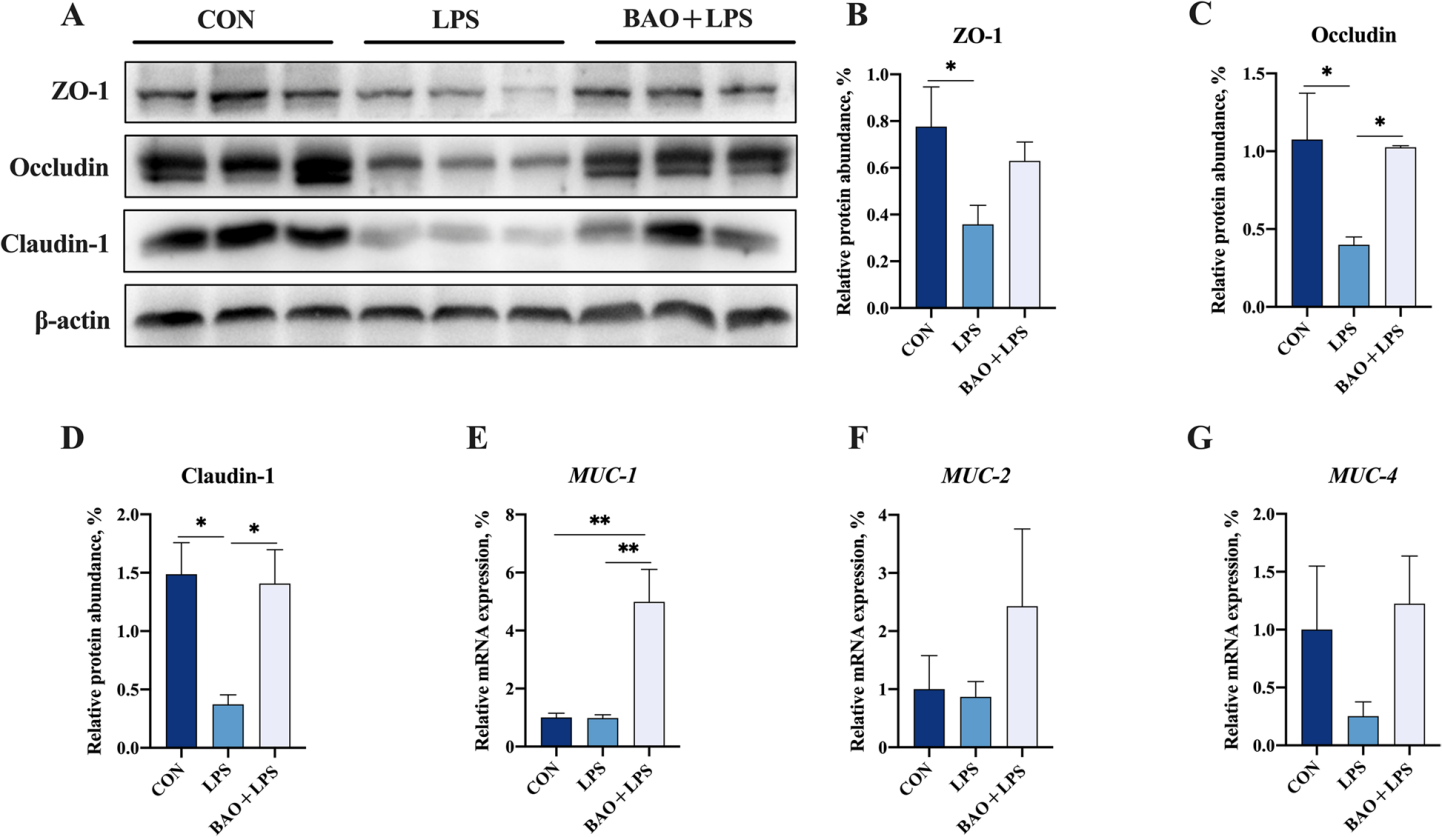
Effects of dietary BAO supplementation on tight junction protein levels and mRNA expression of mucin in the jejunum of weaned pigs challenged with LPS. **A**–**D** The protein expression of ZO-1, Occludin and Claudin-1. **E**–**G** The mRNA expression of *MUC-1*, *MUC-2* and *MUC-4*. Dates are presented as means ± SEM (*n* = 3 for protein expression; *n* = 5 for mRNA expression). ^*^P < 0.05; ^**^P < 0.01. CON, corn-soybean meal basal diets group treated with saline; LPS, corn-soybean meal basal diets group treated with lipopolysaccharide; BAO + LPS, corn-soybean meal basal diets + 5 kg/t benzoic acid + 500 g/t essential oils group treated with lipopolysaccharide

The relative protein abundance of ZO-1, Occludin and Claudin-1 in the jejunum was significantly decreased in the LPS group compared to the CON group (*P* < 0.05, Fig. 4). However, BAO + LPS group showed an increased relative protein expression level of Occludin and Claudin-1 compared with LPS group (*P* < 0.05, Fig. 4). There was no significant difference in the expression of ZO-1, Occludin and Claudin-1 in the jejunum between the CON group and BAO + LPS group (*P* > 0.05, Fig. 4). Moreover, BAO + LPS group showed a significant increase in the mRNA expression of *MUC-1* compared to the CON and LPS groups (*P* < 0.05, Fig. 4). However, there was no significant difference in the expression of *MUC-2* and *MUC-4* between the three groups (*P* > 0.05, Fig. 4).

### Intestinal antioxidant ability

**Fig. 5:**
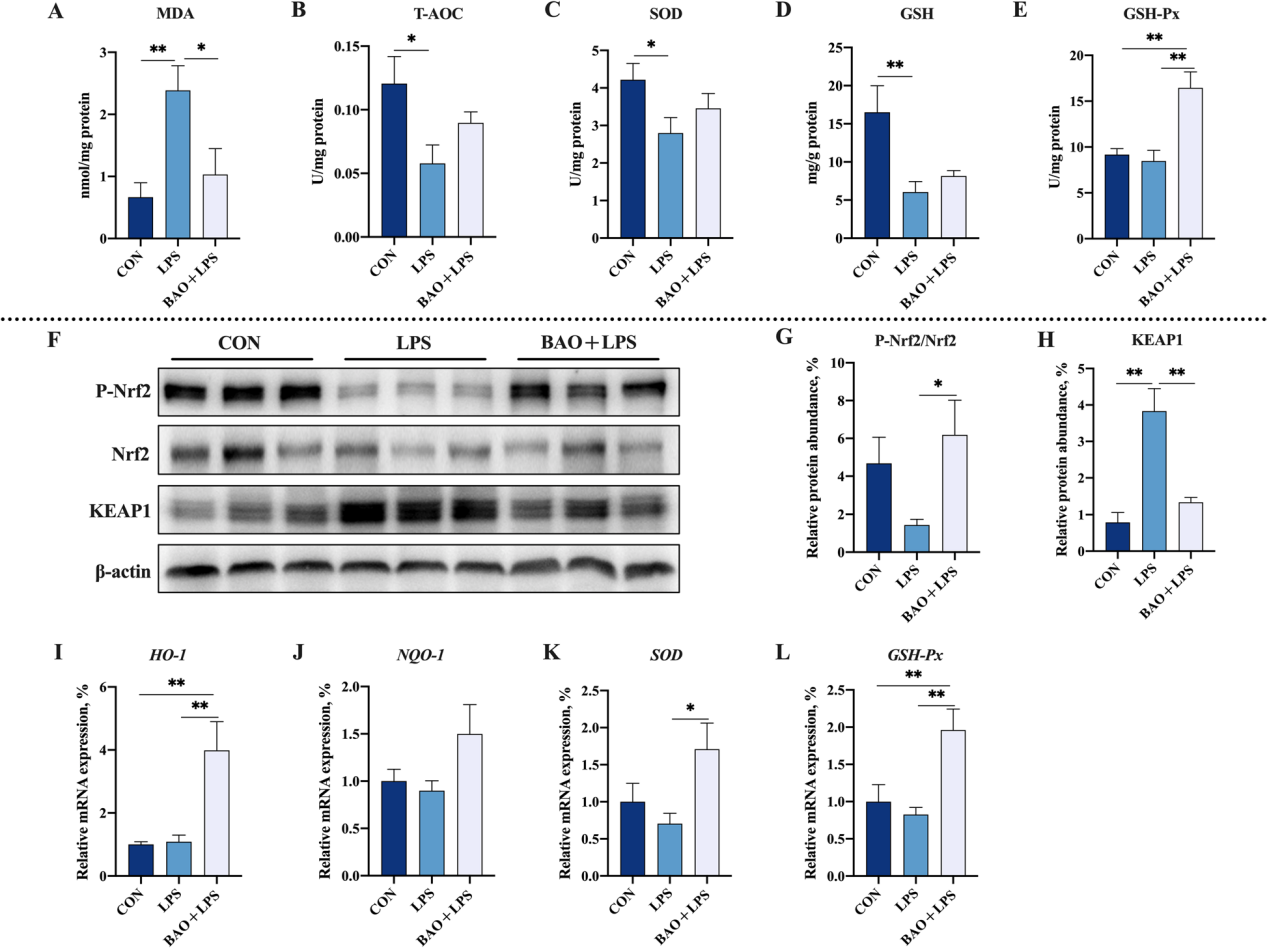
Effects of dietary BAO supplementation on jejunal antioxidant capacity and Nrf2 pathway in weaned pigs challenged with LPS. **A**–**E** The activities of MDA, T-AOC, SOD, GSH and GSH-Px. **F**–**H** The protein expression of P-Nrf2/Nrf2 and KEAP1. **I**–**L** The mRNA expression of *HO-1*, *NQO-1*, *SOD* and *GSH-Px*. Dates are presented as means ± SEM (*n* = 3 for protein expression; *n* = 5 for others). ^*^P < 0.05; ^**^P < 0.01. CON, corn-soybean meal basal diets group treated with saline; LPS, corn-soybean meal basal diets group treated with lipopolysaccharide; BAO + LPS, corn-soybean meal basal diets + 5 kg/t benzoic acid + 500 g/t essential oils group treated with lipopolysaccharide

The content of T-AOC, SOD, and GSH in jejunum was significantly reduced, and the content of MDA was increased in the LPS group compared to the CON group (*P* < 0.05, Fig. 5). However, BAO + LPS group effectively increased level of GSH-Px, and decreased MDA activity compared to the LPS group (*P* < 0.05, Fig. 5). Furthermore, the GSH-Px content in jejunum was significantly increased in the BAO + LPS group compared to the CON group (*P* < 0.05, Fig. 5).

To further investigate the potential mechanisms underlying the antioxidant effects of BAO in the jejunum, we assessed the expression of proteins and genes associated with the Nrf2 pathway. Our results showed that piglets in the LPS group had increased protein level of KEAP1 in the jejunum compared with the CON group (*P* < 0.05, Fig. 5), indicating a downregulation of the Nrf2 pathway. In contrast, piglets in the BAO + LPS group exhibited the decreased protein level of KEAP1 and increased protein level of P-Nrf2/Nrf2 in the jejunum compared with the LPS group (*P* < 0.05, Fig. 5), suggesting an activation of the Nrf2 pathway. Moreover, the protein expression of P-Nrf2/Nrf2 and KEAP1 was not significantly different between the CON and BAO + LPS groups (*P* > 0.05, Fig. 5). Furthermore, the mRNA levels of downstream genes in the Nrf2 pathway, including *HO-1*, *SOD*, and *GSH-Px*, were increased in the BAO + LPS group compared with the LPS group, and the mRNA expression levels of *HO-1* and *GSH-Px* in the BAO + LPS group were even higher than those in the CON group (*P* < 0.05, Fig. 5), indicating a potential protective effect of BAO against oxidative stress in the jejunum.

### Intestinal inflammation level

**Fig. 6:**
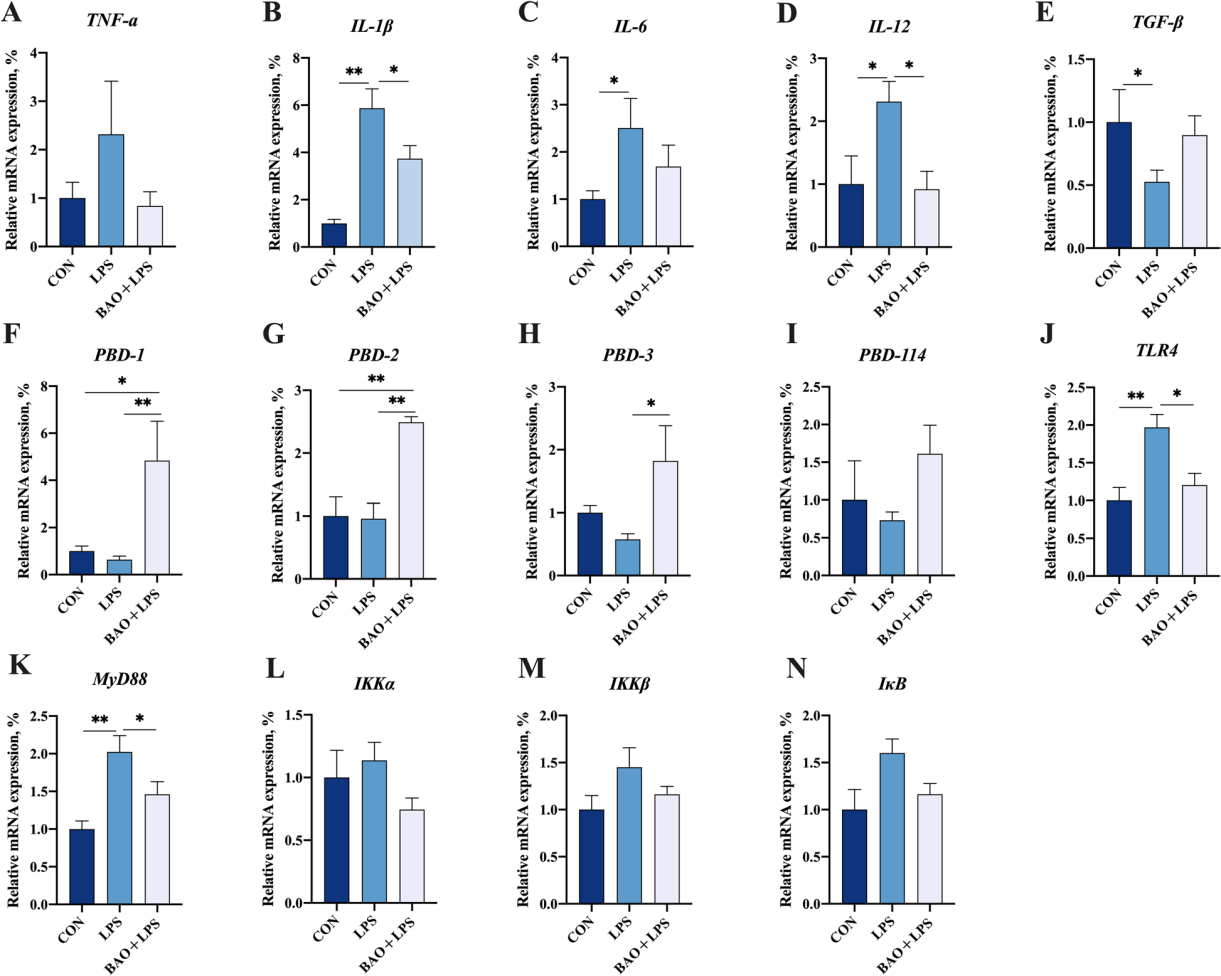
Effects of dietary BAO supplementation on mRNA expressions of inflammation levels in jejunum of weaned pigs challenged with LPS. **A**–**N** The mRNA expression levels of *TNF-α*, *IL-1β*, *IL-6*, *IL-12*, *TGF-β*, *PBD-1*, *PBD-2*, *PBD-3*, *PBD114*, *TLR4*, *MyD88*, *IKKα*, *IKKβ* and *IκB*. Dates are presented as means ± SEM (*n* = 5). ^*^P < 0.05; ^**^P < 0.01. CON, corn-soybean meal basal diets group treated with saline; LPS, corn-soybean meal basal diets group treated with lipopolysaccharide; BAO + LPS, corn-soybean meal basal diets + 5 kg/t benzoic acid + 500 g/t essential oils group treated with lipopolysaccharide

Enhanced mRNA expression level of *IL-1β*, *IL-6*, *IL-12* and reduced mRNA expression level of *TGF-β* in the jejunum of piglets in LPS group were observed compared to the CON group (*P* < 0.05, Fig. 6). Nevertheless, compared to the LPS, significantly downregulation were noticed in *IL-1β* and *IL-12* mRNA abundance in the jejunum of BAO + LPS group (*P* < 0.05, Fig. 6). Also, the relative mRNA expression levels of *PBD-1*, *PBD-2*, and *PBD-3* in the jejunum of BAO + LPS group were superior to LPS group (*P* < 0.05, Fig. 6). Moreover, a critical addition in *PBD-1* and *PBD-2* of mRNA levels was observed in BAO + LPS than CON group (*P* < 0.05, Fig. 6). Compared to the CON group, LPS increased the mRNA expression of *TLR4* and *MyD88*, while BAO reversed the phenomenon of higher expression of *TLR4* and *MyD88* at the mRNA level in the jejunum (*P* < 0.05, Fig. 6).

**Fig. 7:**
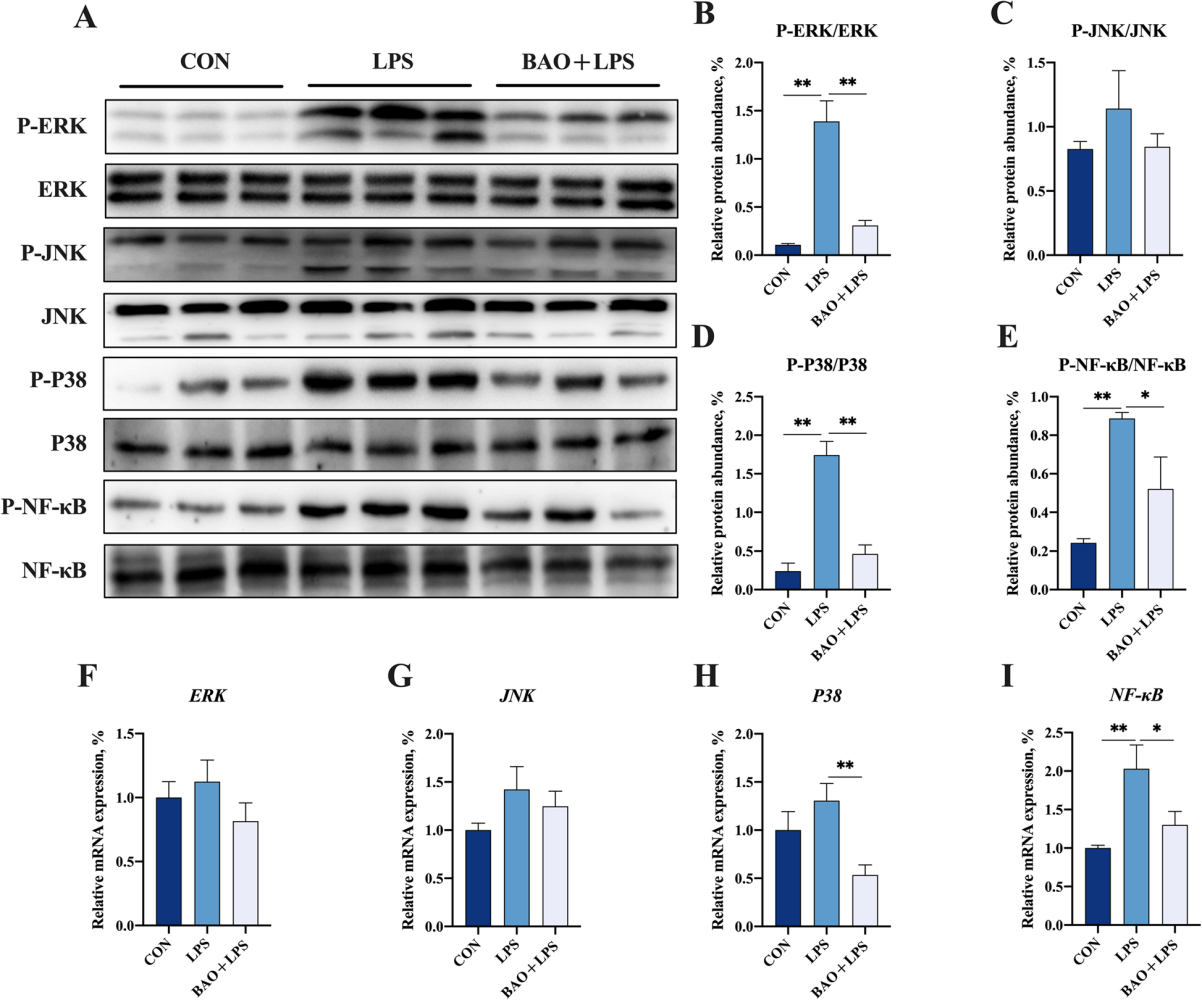
Effects of dietary BAO supplementation on protein and mRNA expressions of MAPK/NF-κB inflammatory pathway in jejunum of weaned pigs challenged with LPS. **A**–**E** The protein expression levels of P-ERK/ERK, P-JNK/JNK, P-P38/P38 and P-NF-κB/NF-κB. **F**–**I** The mRNA expression levels of *ERK*, *JNK*, *P38* and *NF-κB*. Dates are presented as means ± SEM (*n* = 3 for protein expression; *n* = 5 for mRNA expression). ^*^P < 0.05; ^**^P < 0.01. CON, corn-soybean meal basal diets group treated with saline; LPS, corn-soybean meal basal diets group treated with lipopolysaccharide; BAO + LPS, corn-soybean meal basal diets + 5 kg/t benzoic acid + 500 g/t essential oils group treated with lipopolysaccharide

The relative protein abundance of P-ERK/ERK, P-P38/P38 and P-NF-κB/NF-κB in the jejunum of piglets in the LPS group was significantly higher than that in the CON and BAO + LPS groups (*P* < 0.05, Fig. 7). However, dietary BAO significantly reduced the high protein expression of P-ERK/ERK, P-P38/P38 and P-NF-κB/NF-κB induced by LPS (*P* < 0.05, Fig. 7). The relative protein abundance of P-JNK/JNK showed no significant difference among the three groups (*P* > 0.05, Fig. 7). Moreover, BAO + LPS group showed lower mRNA expression levels of jejunal *P38* and *NF-κB* than those in the LPS group (*P* < 0.05, Fig. 7). These results suggest that BAO supplementation can attenuate LPS-induced activation of the *ERK*, *P38*, and *NF-κB* signaling pathways in the jejunum of piglets. The protein expression of P-ERK/ERK, P-JNK/JNK, P-P38/P38, and P-NF-κB/NF-κB and the mRNA expression of *ERK*, *JNK*, *P38*, and *NF-κB* were similar between the CON and BAO + LPS groups (*P* > 0.05, Fig. 7).

### Microbiota populations

**Fig. 8:**
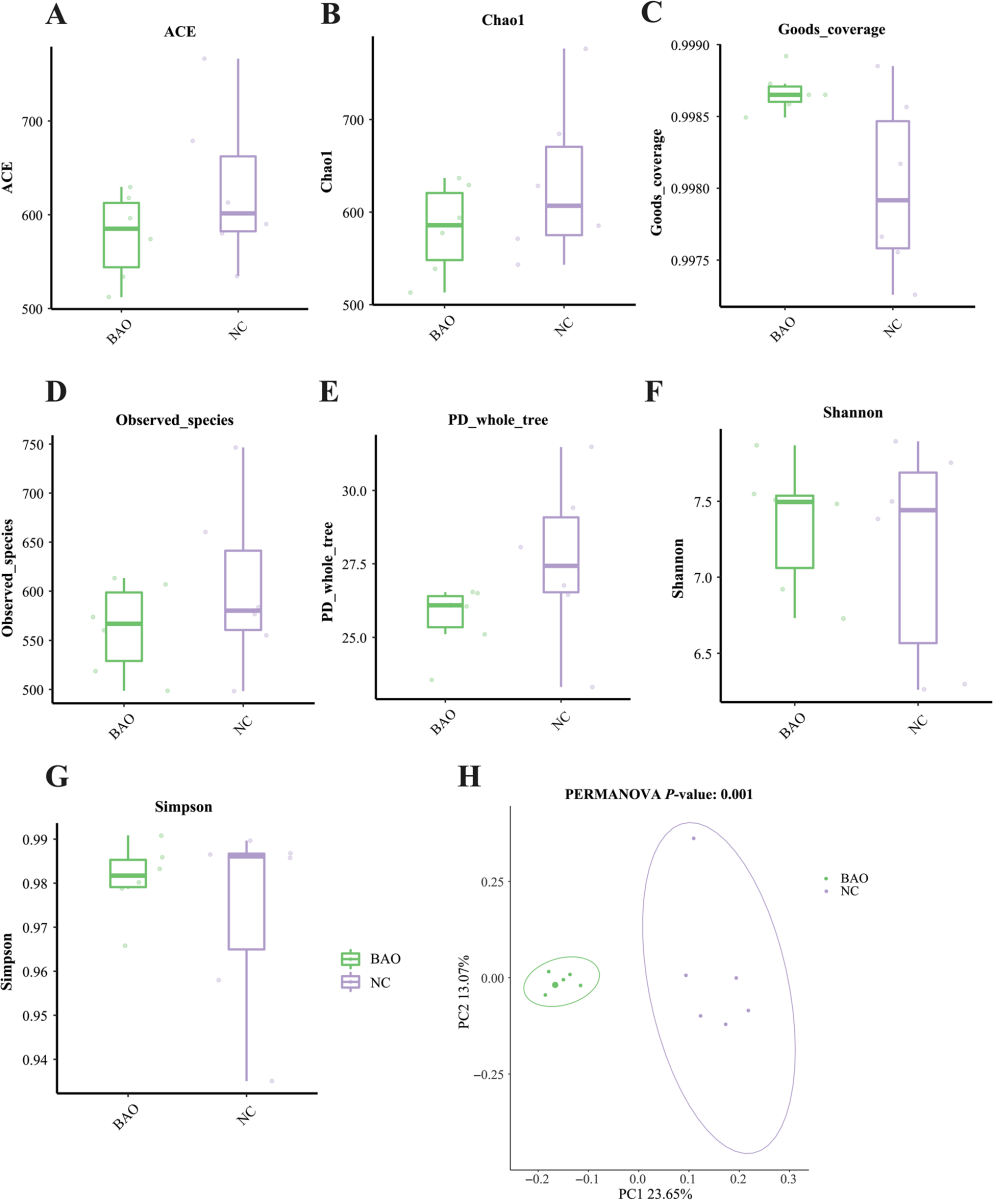
The shifts in fecal alpha and beta diversity of NC and BAO piglets. **A** ACE index of microbiota. **B** Chao1 index of microbiota. **C** Goods_coverage index of microbiota. **D** Observed_species index of microbiota. **E** PD_whole_tree index of microbiota. **F** Shannon index of microbiota. **G** Simpson index of microbiota. **H** Principal component analysis (PCoA) scores plot. Dates are presented as means ± SEM (*n* = 6). ^*^P < 0.05; ^**^P < 0.01. NC, corn-soybean meal basal diets; BAO, corn-soybean meal basal diets + 5 kg/t benzoic acid + 500 g/t essential oils

Figure 8A–G illustrate the alpha diversity analysis, and it was found that there were no significant differences in ACE, Chao1, goods_coverage, observed_species, PD_whole_tree, Shannon, and Simpson indices between the BAO and NC groups (*P* > 0.05), indicating that BAO supplementation did not affect the richness and diversity of the fecal microbiota.

In the beta diversity analysis, PCoA was performed to examine the differences in fecal microbiota composition between the BAO and NC groups. As presented in Fig. 7H, there was a clear separation between the fecal microbiota communities of the BAO and NC groups, indicating that BAO significantly altered the fecal microbiota composition.

**Fig. 9:**
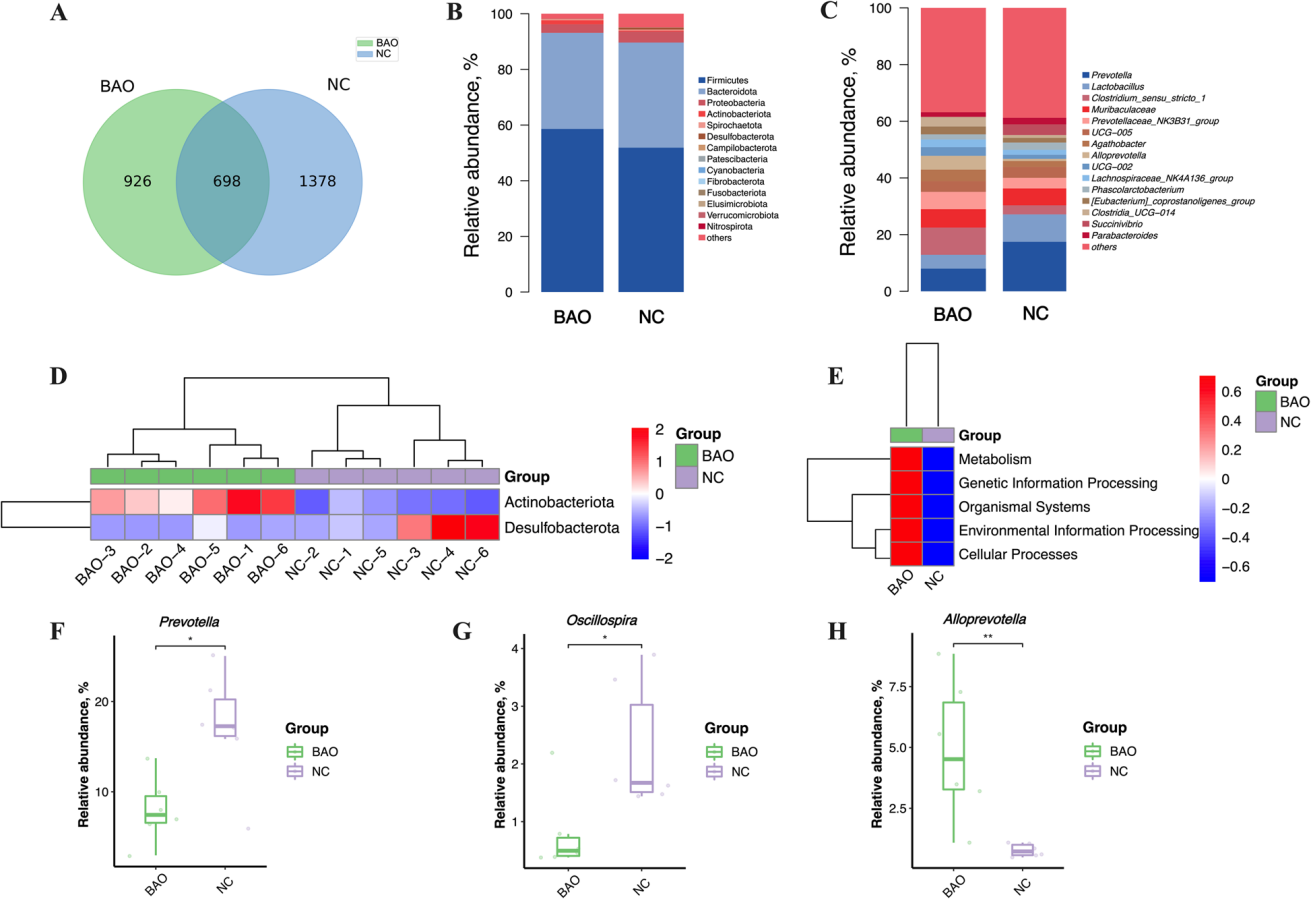
The relative abundance of fecal microbial communities of NC and BAO piglets. **A** Venn diagram. **B** Relative abundance at the phylum level. **C** Relative abundance at the genus level. **D** Heat map of differential bacteria at phylum level. **E** Heat map at KEGG pathway. **F**–**H** The relative abundance of *Prevotella*, *Oscillospira* and *Alloprevotella* at genus level. Dates are presented as means ± SEM (*n* = 6). ^*^P < 0.05; ^**^P < 0.01. NC, corn-soybean meal basal diets; BAO, corn-soybean meal basal diets + 5 kg/t benzoic acid + 500 g/t essential oils

A combined number of 698 shared ASV out of the total ASV overlapped between the NC and BAO group (Fig. 9A). Figure 9B shows the relative abundance of the dominant bacterial phyla, including Firmicutes, Bacteroidetes, Proteobacteria, Actinobacteria, and Spirochaetes. Figure 9C displays the relative abundance of the dominant bacterial genera, including *Prevotella*, *Lactobacillus*, *Clostridum_sensu_stricto_1*, and *Muribaculaceae*. Notable differences were observed in the relative abundance of Actinobacteria and Desulfobacterota between the BAO and NC groups (Fig. 9D). At the genus level (Fig. 9F–H), BAO significantly reduced the relative abundance of *Prevotella*, *Oscillospira* and increased the relative abundance of *Alloprevotella* as compared with the NC group (*P* < 0.05), indicating that BAO supplementation had a significant impact on the gut microbiota composition at the genus level. The enhancement of metabolism, genetic information processing, organismal systems, environmental information processing, cellular processes pathways in the BAO group was found in Fig. 9E.

### Correlation analysis

**Fig. 10:**
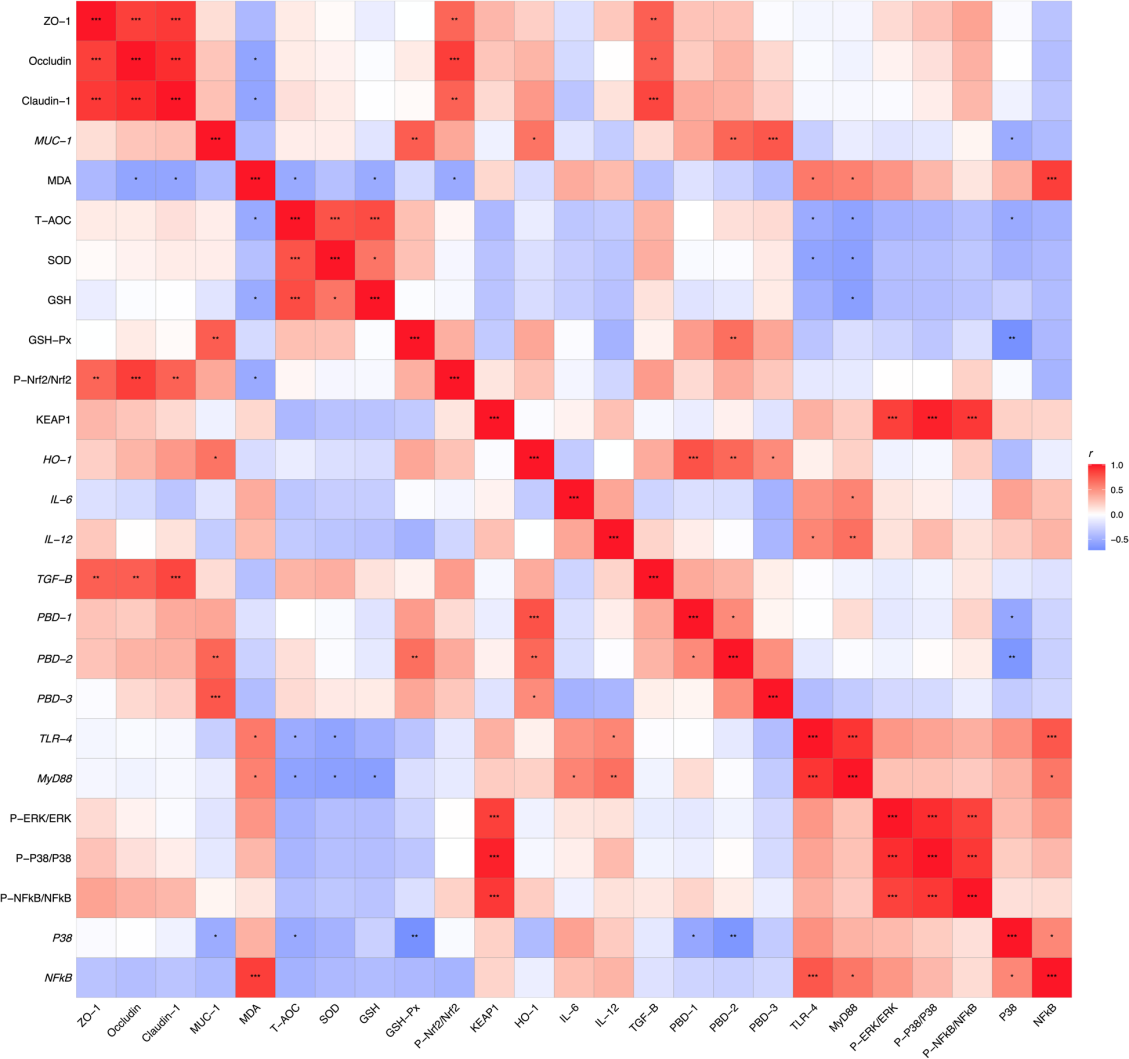
Heatmap of Pearson’s correlation coefficients among intestinal barrier function, antioxidant capacity and inflammation level. In the figure, red with a *P* < 0.05 represents a significant positive correlation, blue with a *P* < 0.05 represents a significant negative correlation, and white represents no correlation. ^*^P < 0.05; ^**^P < 0.01

The correlation analysis of intestinal barrier function, antioxidant capacity and inflammation level are shown in Fig. 10. Intestinal tight junction proteins (ZO-1, Occludin, and Claudin-1) were positively correlated with P-Nrf2/Nrf2 and *TGF-β*, and negatively correlated with MDA. The antioxidant factors (T-AOC, SOD, and GSH) were negatively correlated with *TLR4* and *MyD88*, while MDA was positively correlated with *TLR4* and *MyD88*. Inflammatory cytokines (*IL-6* and *IL-12*) were positively correlated with *TLR4* and *MyD88*. *PBD-1*, *PBD2* and *PBD-3* were positively correlated with MUC-1 and *HO-1*, and negatively correlated with P38. Inflammatory pathways (P-ERK/ERK, P-P38/P38, and P-NFKB/NFKB) was positively correlated with KEAP1.

## Discussion

It is widely recognized that weaning stress in piglets can lead to negative inflammatory reactions, including lower immune function and antioxidant capacity, damaged intestinal structure and function, and disturbed microflora, resulting in reduced feed efficiency and stunted growth performance [22]. Over the last decade, whether BA or EO have been widely used as effective antibiotic substitutes during the weaning process due to BA has the effect of antibacterial, anti-corrosion and stimulating the secretion of intestinal digestive enzymes, and EO has the effect of antioxidant, anti-inflammatory and improving the body's immune capacity, which can improve intestinal health to promote the growth performance and health status of piglets [5, 8, 10]. A growing body of evidence has suggested that appropriate supplementation of either BA or EO can improve ADFI, ADG, F/G ratio, and reduce diarrhea rate in piglets [23, 24]. Recent studies have also shown that the combination of BAO has a stronger synergistic effect than using either substance alone [25]. In this study, despite the relatively short trial time of 14 d, we observed higher final weight and ADG, as well as lower F/G ratio in piglets fed diets with BAO mixtures. These results are consistent with previous findings and further confirm the beneficial effects of BAO on promoting growth performance and improving feed efficiency in weaned piglets.

Internal organs are an important part of the animal body, which can maintain the normal growth and development of the body, resist external environmental changes and pathogen invasion, and the good development of various organs can promote the growth of animals [26]. Organ index can evaluate the growth status, metabolism and function of various animal organs, so as to reflect the development degree of animal organs and physiological status of animals [27]. Liver, kidney and spleen are all very important metabolic organs in animals. If the organ index decreases, it will seriously affect the basic physiological activities of animals. The liver index can evaluate whether the liver function of animals is normal. When the liver is damaged or diseased, the liver index will decrease significantly [28]. When the health of the animal body is improved, the liver index will be significantly increased [29]. Previous studies have shown that dietary addition of BAO can improve liver organ index [30]. Similarly, our results show that compared with CON group, piglets in BAO + LPS group have higher liver index, which proves that dietary addition of BAO has a protective effect on internal organs and is more conducive to the healthy growth of weaned piglets.

As a constituent of the outer membrane of Gram-negative bacteria, LPS is widely recognized as a primary inducer of the intestinal immune response, leading to the production and release of pro-inflammatory cytokines, which can result in systemic inflammation and dysregulation of oxidative stress, as well as structural and functional damage to the intestine [31]. The intraperitoneal injection of LPS challenge has been a widely utilized and effective method to establish pig models of inflammation and intestinal injury, serving as a tool to mimic weaning stress and bacterial infection [32, 33]. Compared with oral LPS which is digested and absorbed into the body, intraperitoneal injection of lipopolysaccharide can play a role in a short time and enter the blood faster and more directly [34]. Once the immune cells in the blood encounter LPS, they will think that there are pathogens in the place where they should not enter, and the body will have a strong inflammatory response [35]. Therefore, intraperitoneal injection of LPS is more suitable for constructing acute inflammation model. Plasma antioxidant and immune levels are reliable indicators for assessing the systemic health status of weaned piglets. It is generally recognized that the antioxidant systems, comprising of SOD, GSH, GSH-Px, MDA, and T-AOC, are crucial in maintaining the homeostasis of oxidative stress in the body, and play a critical role in the defense mechanism against oxidative damage [36, 37]. Proinflammatory cytokines, such as TNF-α, IL-1β, IL-6, and IL-12, which exert a significant influence in regulating host immunity, are rapidly generated and secreted by immune cells in response to various adverse external stimuli, and their expression levels are closely associated with the systemic health status of the body under stress conditions [38, 39]. Previous studies have shown that injection of LPS in weaned piglets leads to a decrease in serum levels of T-AOC and SOD, but dietary supplementation with OA and EO can mitigate this effect and increase the levels of T-AOC and GSH-Px. [40, 41]. In addition, LPS stimulation induces systemic inflammation and the expression of pro-inflammatory factors, such as TNF-α, IL-6, and IL-1β, which can be reduced by piglet dietary supplementation with BA and oregano EO, as observed in the study by Pu et al. [42]. These findings suggest that the dietary supplementation of OA and EO may have the potential to alleviate oxidative stress and inflammation in weaned piglets, thereby promoting post-stress recovery and highlighting their potential reparative effects. In the present study, it was observed that the group of piglets fed with a diet supplemented with BAO exhibited a superior systemic antioxidant capacity and inflammatory profile. It is noteworthy, however, that in this experiment, the piglets were subjected to LPS challenge after a 14-d BAO supplementation period, implying that the observed effects of BAO treatment may be attributed to its potential to enhance the health status of the piglets, thereby conferring improved stress resilience.

Preserving the integrity of the intestinal tract is paramount for optimal animal health, as the intestine serves as the body's first line of defense against external damage. Impairment of the intestinal barrier can permit the entry of harmful agents, including bacteria, toxins, or pathogens, into the intestine, resulting in the activation of an inflammatory response and the development of a range of health problems [43, 44]. The intestinal barrier is composed of various physical components, including the intestinal epithelial cells which form a tight layer for the selective passage of molecules and ions, and the mucus layer acting as a primary defense against pathogenic microbes [45]. Our results indicated that piglets exposed to LPS suffered extensive damage to their intestinal structures, as evidenced by a decrease in VH and VH/CD and an increase in CD in the duodenum, jejunum, and ileum, which is consistent to previous study [46]. Moreover, the intestinal barrier was significantly compromised by the LPS attack, as indicated by the marked downregulation of ZO-1, Occludin, and Claudin-1. However, we observed that dietary supplementation of BAO effectively alleviated LPS-induced intestinal injury, as demonstrated by the significant improvement in intestinal morphology indicators, such as VH and VH/CD ratio in the duodenum, jejunum, and ileum, as well as the enhancement of intestinal barrier function, which was reflected by the restoration of tight junction proteins (Claudin-1, Occludin) and *MUC-1* expression. The findings suggest that prior dietary supplementation of BAO can effectively enhance the physical barrier and structural stability of the intestinal tract, resulting in an improved capacity of piglets to withstand weaning stress, which is likely attributable to the observed decrease in systemic inflammatory levels.

After weaning, piglets suffer from changes in the environment and diet from liquid feed to solid powder feed, often leading to an imbalance in the intestinal redox system and excessive reactive oxygen species, which can damage the physiological function of the intestinal tract [47, 48]. Improving intestinal antioxidant capacity can help weaned piglets cope with weaning stress, which in turn promotes growth performance and health status. It has been reported that LPS treatment leads to disordered intestinal oxidative stress and downregulated expression of antioxidant enzymes in the intestine [49], which was similarly observed in our present study. In addition, our findings suggest that dietary supplementation with BAO has the potential to alleviate oxidative stress and rebalance the redox system in the intestine, as demonstrated by increased GSH-Px activity and decreased MDA abundance. Under normal physiological conditions, the transcription factor Nrf2 forms a complex with its negative regulator Keap1, which is sequestered in the cytoplasm [50, 51]. In response to oxidative stress, excessive ROS dissociates KEAP1 from Nrf2, leading to the transfer of phosphorylated Nrf2 to the nucleus, where it induces the expression of downstream genes such as *HO-1*, *NQO-1*, and *SOD*, to eliminate ROS [52]. Previous research has demonstrated that the Nrf2 signaling pathway is often inhibited in pigs stimulated with LPS, while dietary supplementation with BA or EO has been shown to improve the intestinal oxidative status of piglets via the Nrf2 pathway [53]. Consistent with these findings, our study observed that LPS injection resulted in increased levels of KEAP1 protein expression in jejunal mucosa. Conversely, BAO supplementation significantly downregulated KEAP1 protein expression, upregulated P-Nrf2/Nrf2 protein expression, and improved mRNA expression of *HO-1*, *SOD*, and *GSH-Px* in the jejunum of LPS-challenged weaned pigs. These results suggest that dietary supplementation with BAO enhances the intestinal antioxidant capacity in LPS-challenged weaned pigs, possibly by increasing the activity of antioxidant enzymes through activation of the Nrf2/KEAP1 pathway. In addition, the limited 2-week duration of dietary intervention is indeed a pertinent factor to consider. The relatively short 2-week dietary intervention period, coupled with the inherent vulnerability of the intestine during weaning, may have constrained BAO’s ability to fully manifest its protective effects in response to LPS stimulation. Consequently, while certain relevant indicators exhibited significant improvements, others did not display substantial changes.

The dysregulation of intestinal antioxidant capacity is commonly concomitant with the development of inflammation in piglets under weaning stress. As expected, the administration of LPS enhanced mRNA concentrations of *IL-1β*, *IL-6*, *IL-12* in jejunum and caused a significant diminish in jejunal mRNA concentration of *TGF-β*, while dietary BAO significantly reduced the addition of *IL-1β* and *IL-12* induced by LPS. Porcine β-defensin (PBD), an endogenous antibiotic with anti-inflammatory response of the body, significantly relieves LPS-induced inflammatory response and injury by reducing the secretion of pro-inflammatory cytokines and apoptosis [54]. Our data found that the mRNA expression of *PBD-1*, *PBD-2* and *PBD-3* was enhanced in the BAO + LPS piglets compared with LPS piglets, suggesting that supplementation of BAO can ease LPS-induced intestinal inflammation in weaned piglets by inhibiting the production of inflammatory factors. LPS induces the production of inflammatory factors and exacerbates the inflammatory response in intestinal mucosa by activating the TLR4 mediated NF-κB and MAPK inflammatory pathways [55]. TLR4 is a key receptor mediated by inflammation and plays a special role in maintaining intestinal homeostasis by recognizing and specifically binding LPS to regulate inflammatory response [56]. The NF-κB and MAPK signaling pathways play an important role in regulating inflammatory responses induced by external stimuli, regulating downstream targets and promoting the release of pro-inflammatory cytokines when activated, ultimately leading to tissue damage [57]. Previous experiments have observed that *Escherichia coli* stimulation can enhance the mRNA expression of *TLR4*, *MyD88*, *P38* and *NF-κB*, while OA and EO supplementation can inhibit the mRNA expression of *NF-κB* [42]. In the present study, increased mRNA abundance of *TLR4*, *MyD88*, *NF-κB* and the protein concentrations of P-ERK/ERK, P-P38/P38 and P-NF-κB/NF-κB was also found after LPS challenge, but dietary supplementation with BAO significantly weakened the mRNA abundance of *TLR4*, *MyD88*, *P38*, *NF-κB* and protein concentrations of P-ERK/ERK, P-P38/P38 and P-NF-κB/NF-κB compared with LPS piglets, which further suggests that BAO supplementation improved the capacity of weaned pigs to resist the intestinal inflammation by inhibiting TLR4/MAPK/NF-κB pathways to reduce the expression of intestinal inflammatory factors.

The intestinal microbiota plays a crucial role in safeguarding the gut from damage and promoting normal growth and health through its metabolic and immune functions [58]. EO and OA have been found to possess potential antibacterial properties and improve intestinal homeostasis by inhibiting the proliferation of harmful microorganisms and promoting the growth of beneficial bacteria [59, 60]. Our alpha diversity analysis did not reveal any significant differences in intestinal microbiota evenness and richness between the BAO and NC groups. However, our PCoA plots showed distinct separation between the two groups, indicating that the microbial structure and richness of weaned piglets were affected by the addition of BAO to their diet. These findings are consistent with the results of Ma et al. [40], who demonstrated that the addition of EO and OA to weaned piglet diets can alter microbial beta diversity. Our results also revealed that the most dominant phyla in the fecal samples of the BAO and NC groups were Firmicutes and Bacteroidetes, which is in line with prior investigations [61]. At the phylum level, we observed an increased abundance of Actinobacteriota and a decreased abundance of Desulfobacterota in the intestinal microbiota of piglets in the BAO group. Studies have shown that Actinobacteriota produce antibiotics, immunomodulatory compounds, and metabolites that possess antimicrobial and antifungal actions [62]. Rao et al. [63] also found that reducing the abundance of Desulfobacterota can improve intestinal bacterial imbalance, suppress intestinal inflammation, and enhance intestinal barrier function, as evidenced by elevated expression of ZO-1 and Occludin, which is consistent with our results. Furthermore, our results showed a lower relative abundance of *Prevotella*, *Oscillospira* and a higher abundance of *Alloprevotella* at the genus level in the BAO group. The *Prevotella* genus is known to promote inflammation and the development of colorectal cancer [64], which supports our finding that BAO can protect the intestine from inflammation. In addition, *Oscillibacter* has been reported to be a key enteric pathogenic bacterium associated with the onset of enteric colitis [65]. On the other hand, *Alloprevotella* is considered to be a potentially beneficial bacteria in the gut and is closely associated with the production of short-chain fatty acids such as succinates and acetates [66–68]. Interestingly, the abundance of *Prevotella* was negatively associated with weight fluctuations, while the abundance of *Alloprevotella* was positively associated with weaning weight [69], providing further evidence that BAO can increase ADG in weaned piglets.

## Conclusion

In conclusion, the current study provides robust evidence supporting the beneficial effects of BAO on growth performance, feed efficiency and modulating the composition of the microbiota in weaned piglets. Furthermore, our innovative experimental design, where BAO was administered prior to LPS challenge, highlights the potential of BAO in enhancing piglets' anti-stress ability by promoting intestinal structural integrity, balancing oxidative stress and inflammation. These findings highlight the potential of BAO as a natural feed additive to promote piglet health and growth and provide new insights into the mechanisms underlying its beneficial effects.

### Supplementary Information

**Additional file 1: Table S1. **Composition of the diet (as-fed basis)**.**

## Data Availability

The data analyzed during the current study are available from the corresponding author on reasonable request.

## Abbreviations

ADFI: Average daily feed intake
ADG: Average daily gain
BA: Benzoic acid
BAO: Benzoic acid and essential oils
CD: Crypt depth
EO: Essential oils
F/G: Feed intake/body gain
GSH: Reduced glutathione
GSH-Px: Glutathione peroxidase
IL: Interleukin
LEfSe: Linear discriminant analysis effect size
LPS: Lipopolysaccharide
MDA: Malonaldehyde
Nrf2: Nuclear factor erythroid 2-related factor
PCoA: Principal coordinate analysis
PVDF: Polyvinylidene difluoride
SEM: Standard error of the mean
SOD: Superoxide dismutase
T-AOC: Total antioxidant capacity
TNF-α: Tumor necrosis factor-α
VH: Villi height
