# Supplementary material for: Dietary supplementation of benzoic acid and essential oils combination enhances intestinal resilience against LPS stimulation in weaned piglets
Source: J Anim Sci Biotechnol. 2024 Jan 19;15:4. doi: 10.1186/s40104-023-00958-6 (PMC10797991; doi:10.1186/s40104-023-00958-6)
Supplement: Supplementary file 1 — Additional file 1: Table S1. Composition of the diet (as-fed basis). [file 40104_2023_958_MOESM1_ESM.docx]

**Table S1** Composition of the diet (as-fed basis)

| Ingredients | Content, % |
| --- | --- |
| Corn | 62.30 |
| Soybean meal | 16.00 |
| Fermented soybean meal | 6.50 |
| Extruded soybean | 7.00 |
| Soy protein isolate | 1.30 |
| Soyabean oil | 2.00 |
| Limestone | 0.80 |
| Salt | 0.40 |
| L-lysine-HCl, 78% | 0.45 |
| L-methionine | 0.15 |
| L-threonine | 0.13 |
| L-isoleucine | 0.10 |
| L-tryptophan | 0.01 |
| L-histidine | 0.01 |
| CaHPO_4_ | 1.80 |
| Calcium propionate, 50% | 0.05 |
| Premix^a^ | 1.00 |
| Total | 100.00 |

^a^Provide the following per kg complete diet: Vitamin A, 12,000 IU; Vitamin D_3_, 4,000 IU; Vitamin E, 20 IU; Vitamin K_3_, 5 mg; Vitamin B_1_, 2 mg; Vitamin B_2_, 5 mg; Vitamin B_6_, 6 mg; Vitamin B_12_, 0.03 mg; Niacin, 30 mg; Pantothenic acid, 17 mg; Folic acid, 2.5 mg; Biotin, 0.1 mg; Choline chloride, 500 mg; Fe (as ferrous sulfate), 100 mg; Cu (as copper sulfate), 7 mg; Mn (as manganese sulfate), 5 mg; Zn (as zinc sulfate), 100 mg; I (as calcium iodate), 0.2 mg; Se (as sodium selenite), 0.3 mg
